# Automated Dielectrophoretic Tweezers-Based Force Spectroscopy System in a Microfluidic Device

**DOI:** 10.3390/s17102272

**Published:** 2017-10-04

**Authors:** Min Hyung Kim, Jeongjick Lee, Kihwan Nam, In Soo Park, Myeonggu Son, Hyunchul Ko, Sangyoup Lee, Dae Sung Yoon, Woo-Jin Chang, Sei Young Lee, Young Ro Yoon, Sang Woo Lee

**Affiliations:** 1 Department of Biomedical Engineering, Yonsei University, Wonju 220-710, Korea; kmh900710@gmail.com (M.H.K.); wlslrbsl1@naver.com (J.L.); pakinsoo@hotmail.com (I.S.P.); s92624452@gmail.com (M.S.); kojel84@gmail.com (H.K.); syl235@yonsei.ac.kr (S.Y.L.); 2 Biomedical Research Institute, Korea Institute of Science and Technology, Seoul 136-791, Korea; kihwannam@gmail.com (K.N.); sangyoup@kist.re.kr (S.L.); 3 Department of Biomedical Engineering, University of Science and Technology, Daejeon 305-350, Korea; 4 Department of Bio-Convergence, Korea University, Seoul 136-701, Korea; dsyoon@korea.ac.kr; 5 Mechanical Engineering Department, University of Wisconsin-Milwaukee, 3200 N Cramer St., Milwaukee, WI 53211, USA; wjchang@uwm.edu

**Keywords:** dielectrophoresis, force spectroscopy, force loading rate, intermolecular weak binding interactions

## Abstract

We reported an automated dielectrophoretic (DEP) tweezers-based force spectroscopy system to examine intermolecular weak binding interactions, which consists of three components: (1) interdigitated electrodes and micro-sized polystyrene particles used as DEP tweezers and probes inside a microfluidic device, along with an arbitrary function generator connected to a high voltage amplifier; (2) microscopy hooked up to a high-speed charge coupled device (CCD) camera with an image acquisition device; and (3) a computer aid control system based on the LabVIEW program. Using this automated system, we verified the measurement reliability by measuring intermolecular weak binding interactions, such as hydrogen bonds and Van der Waals interactions. In addition, we also observed the linearity of the force loading rates, which is applied to the probes by the DEP tweezers, by varying the number of voltage increment steps and thus affecting the linearity of the force loading rates. This system provides a simple and low-cost platform to investigate intermolecular weak binding interactions.

## 1. Introduction

The characterizations of biological binding forces are important for understanding biological processes (i.e., cell-cell adhesion, the binding of ligands to receptors, and the folding of protein/RNA/DNA) [1]. In the last two decades, the two most popular techniques, atomic force microscopy (AFM) [2], and optical tweezers [3] have been intensively used to investigate these biological binding forces. In the case of AFM, the approach of a sharp tip functionalized by molecules to a surface functionalized by complementary molecules results in binding forces between two different molecules, and the retraction of the tip from the surface results in the rupture of the force. The rupture force is characterized by these AFM movements. On the other hand, optical tweezers, which use light exerting photon energy on a functionalized particle to manipulate the particle, is brought close to a complementary functionalized surface in order to create the binding force. Through the observation of the behavior of the particle, the force between the functionalized particle and the functionalized surface is characterized. Both techniques are very powerful and are utilized in various applications. For example, AFM can measure the biological binding force with a single pico-newton (pN) resolution, as well as sub-nanometer accurate position controllability in both physiological and non-physiological conditions [4,5,6,7,8]. Optical tweezers can be applied to external forces at 100 pN on various particle sizes (from nanometers to micrometers) when concurrently measuring the three-dimensional displacement of the particles [1,9,10,11]. However, both techniques are serial in nature and provide binding information on a single (or a few) events. Hence, many trials would be required to obtain statistically reliable measurement data. Moreover, in general, the force loading rate applied for the measurement of the biological binding force is limited to the ranges of the techniques of interest (AFM ~10–10^7^ pN/s, and optical tweezers ~10^−1^–10^2^ pN/s) due to low-frequency drift from mechanical or optical components [1,12]. It is very difficult to study the biological binding force as a model system, which applies a wide force loading range from a quasi-equilibrium state (extremely low force loading rate) to a non-equilibrium high pulling event in the same environment at the same time. Furthermore, bringing the AFM probe tip to the right position, and focusing on a particle with a laser beam in order to manipulate the particle are not easy. Another interesting method of investigating biological binding force is using the fluid itself in a microfluidic device. In this technique, the biological binding is generated between particles and a substrate in the microfluidic device. After that, the fluid is applied into the binding system as varying the velocity of it, resulting in the movement of the particles. Associating the movement of the particles with the velocity of the fluid, the biological binding force can be measured [13,14]. This fluidic technique can measure a substantial amount of the binding force at the same time under the same environment so that it can achieve simple, efficient, and high-throughput measurement, compared with the AFM and optical tweezers described above. However, it is difficult to obtain a single pN resolution for the investigation of the binding interaction, and manipulate the particles in there-dimensional displacement. In addition, it is also hard to precisely control the force loading rate. Recently, Lee and Emaminejad [12,15,16,17,18,19,20,21,22] reported that DEP tweezers-based force spectroscopy (DFS) is a useful tool for measuring the biological binding force in order to solve the above issues. DFS can measure numerous binding forces in the same environment simultaneously, because the hundreds of functionalized polystyrene particles (~600 events/mm^2^) act as probes inside the microfluidic device with fabricated interdigitated (IDT) electrodes. Furthermore, this technique allows a wide force loading rate from 10^−4^ to 10^4^ pN/s. The loading rate can be measured and compared to the biological binding force in a non-equilibrium high-pulling experiment or in quasi-equilibrium unbinding events. However, even though these advantages over AFM and optical tweezers techniques exist, DFS measurement systems are still manually controlled with increasing voltage steps that affect the linearity at the same force loading rate, taking optical images that are used to observe the movement of the particles, and applying a voltage that responds to the amplitude of the DEP force loading rate in an arbitrary functional generator. These manually controlled methods might enlarge measurement errors and make accessing the DFS measurement system more complex.

In this study, we improved the previous DFS system by developing an automatic system using the graphic programming environment LabVIEW. We controlled a high-speed charge CCD camera and an arbitrary function generator, resulting in an automatic DFS system based on LabVIEW. This system is allowed to automatically control parameters such as the number of voltage step intervals, the image capture rate, and the applied alternate current (AC) voltage of the force loading rate experiment. In addition, to confirm the synchronization between the measurement module consisting of probes and IDT electrodes and the observation module consisting of the high-speed CCD camera, we compared the AC signal increment rate applied to the IDT electrode with an image capture rate of the camera using a light-emitting diode (LED). After that, we also verified the reliability of the automatic DEP system by comparing the measurement data obtained by the new system and the data presented in the previous report. Furthermore, we characterized the linearity of the applied signal in the automatic system through experiments on biological interactions (such as electrostatic repulsion and hydrogen bonding) by applying various voltage increment step intervals under the same force loading rate.

## 2. Materials and Methods

### 2.1. System Structure

**Figure 1:**
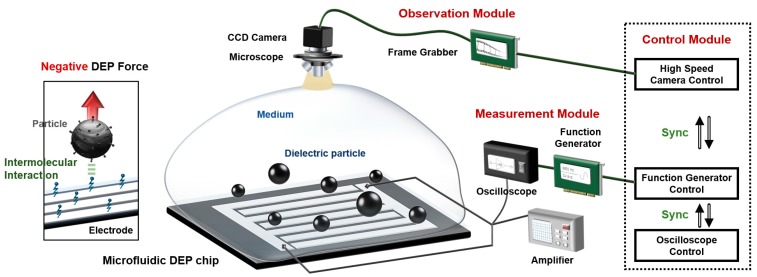
System diagram of the dielectrophoretic (DEP) tweezers-based force spectroscopy (DFS) system. The DFS system consists of three modules: an observation module, a measurement module, and a control module. In the control module, three components manipulate each device in the observation and measurement modules with synchronization.

Figure 1 shows the schematic diagram of the automatic DFS system. The measurement module is composed of three parts: (1) a microfluidic device that includes polystyrene-functionalized particles used as probes and IDT electrodes used as DEP tweezers, along with an arbitrary function generator (NI PCI-5421, National Instrument, Austin, TX, USA) connected to an amplifier that applies voltage to the IDT electrodes to generate a DEP force (WMA-300, Falco Systems, Amsterdam, The Netherlands) and an oscilloscope that monitors the frequency and amplitude of the applied voltage in real time (Wavesurfer 432, Teledyne Lecroy Corp., Chestnut Ridge, NY, USA) (measurement module); (2) in the observation module, microscopy connected to a high-speed CCD camera that observes the particles’ movement that works to save the image captured from the camera in order to control the rate, size, and type of the capture image (Motion Scope M3, Integrated Design Tools, Inc., Tallahassee, FL, USA) and a frame grabber (NI PCIe-1429, National Instrument, Austin, TX, USA) (observation module); (3) tools (such as the function generator, the frame grabber, and the oscilloscope) that are controlled by a control module consisting of a high-speed CCD camera control section, a function generator section, and an oscilloscope control section. Each section is coded by a LabVIEW program (LabVIEW 2012, National Instrument, Austin, TX, USA) (control module).

### 2.2. Program Structure of the Automatic System for DFS

**Figure 2:**
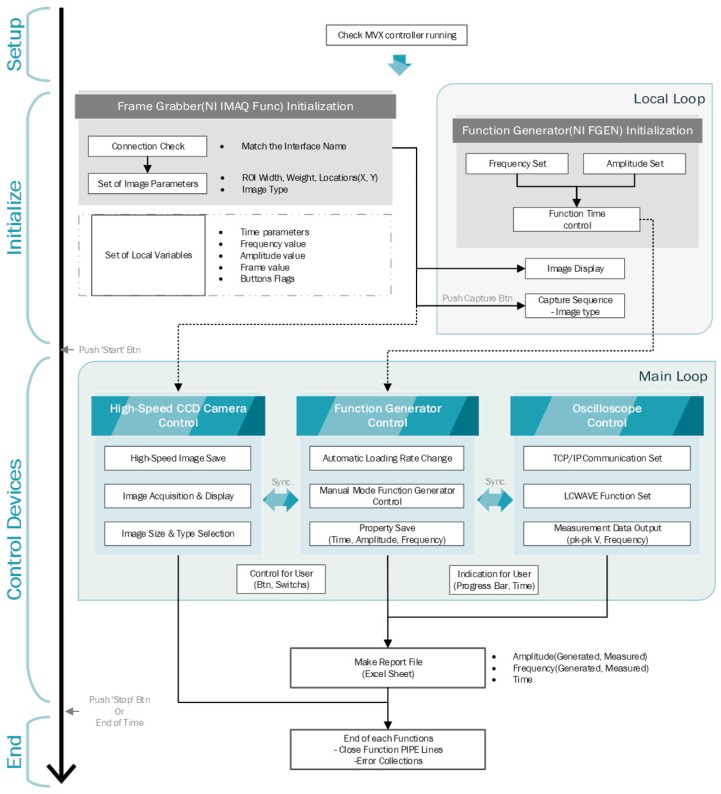
Program structure of the automatic system for DFS using LabVIEW: This structure consists of four sections: the Setup, Initialize, Control Devices, and End sections. Each section describes the role of the devices in the measurement and observation modules, as shown in Figure 1.

The program structure to control the DFS system in our customized LabVIEW program consists of four sections, including the setup, initialization, device controller, and exit sections, as shown in Figure 2. Each section is described in Figure 2. (1) In the setup section, the program checks whether ‘MVX controller running’, which should be run to control the high-speed CCD camera using LabVIEW, is running or not. (2) The frequency and amplitude of the AC signal applied from the arbitrary function generator are applied to the region of interest (ROI) width, height, and image type in the frame grabber, and local variables are set as described in the initialization section. When the program initializes, the image of the microfluidic chip from the high-speed CCD camera in real time is shown. Meanwhile, the experimental time corresponding to the loading rate is calculated. (3) The operating functions of the frame grabber, the arbitrary function generator, and the oscilloscope are controlled by the control device section. The “High-Speed CCD Camera Control” takes hold of the frame grabber, which manipulates the images captured by the high-speed CCD camera. In order to vary the parameters of the applied AC signal, the “Function Generator Control” is used. An automatic loading rate increment or decrement with constant intervals is also restrained in this part. The last component of this control device section is “Oscilloscope Control.” The measured data from the oscilloscope are displayed and saved by communicating with the Transmission Control Protocol (TCP) and Internet Protocol (IP) sockets. In the end section, the data for the applied voltage and frequency, which are generated by the arbitrary function generator and measured by the oscilloscope, are saved to the Excel file, and the program is stopped. In order to precisely measure the rupturing moment of the intermolecular bond in this system, synchronization of the applied AC voltage and image capture by the high-speed CCD camera are extremely important. To verify this property, an LED circuit connected to the function generator was observed under the camera. Step signals from the function generator were applied to the circuit at varying frequencies, and LED images were captured by the high-speed CCD camera with the frames corresponding to the AC frequencies.

### 2.3. Fabrication Process of the DFS Chip and Sample Preparation

An IDT electrode array pattern with a 0.2 μm thick chromium layer was fabricated on an oxidized silicon wafer using a thermal evaporator and lift-off process, where each electrode in the array pattern is 40 μm wide and 10 μm apart. Silicon dioxide was deposited on the IDT electrodes (0.8 μm thick) as an insulator using plasma enhanced chemical vapor deposition with a tetraethylorthosilicate (TEOS) source. Then, the contact pads that are used to apply an AC voltage to the IDT array were opened by a wet etching process using a buffered oxide etch (BHF). In order to functionalize the oxide surface, it was first cleaned by sequentially using a piranha solution (H_2_SO_4_:H_2_O_2_ (1:1)) for 20 min and solvent solutions (acetone and methanol). After that, the carboxyl-terminated oxide surface was functionalized using 100 mM 3-(triethoxysilyl)propylsuccinic anhydride (TESPSA, Gelest, Morrisville, PA, USA) in an organic solvent (toluene, 99.8%). After 24 h, the micro-chip was rinsed with toluene, *N*,*N*-dimethylformamide (DMF), and DI water for 5 min each. The chip was then dried by gently blowing N_2_ on it. After functionalizing the oxide surface in the micro-chip, a polydimethylsiloxane (PDMS) layer was attached to the chip to make an open reservoir. Fifteen-micrometer carboxyl-terminated functionalized polystyrene particles (Kisker Biotech GmbH & Co. KG, Steinfurt, Germany) suspended in a DI water (~4 × 10^4^ particles/mL) solution were introduced into the reservoir, and a glass slide was used to cover the top of it. For the formation of electrostatic repulsion and hydrogen bonding between the oxide surface and particles, the pH of the suspended solution was adjusted with hydrochloric acid (HCl) at 7 and 3.8 in the experiment, respectively [13,14,16]. Despite the electrostatic repulsion was generated between the oxide surface and beads at pH 7, a Van der Waals interaction was formed when the beads closely approached the oxide surface [14]. Therefore, in the experiment using the automatic DFS system, Van der Waals interactions and hydrogen bonding were investigated.

### 2.4. Operation of the DEP Force

First of all, to generate the DEP force for measuring the intermolecular binding force in the micro-chip, a sinusoidal signal at 1 MHz from the function generator was applied into the electrode. When the high amplitude of the applied signal was needed for the experiment, an amplifier was connected to the function generator. All parameters of the applied voltage signal were set up using the graphical user interface (GUI) panel shown in Figure 1, and digitized voltage increment steps correlated to the linear ramp of the force loading rate were also controlled with this system. For example, when the loading rate was 4 pN/s, the magnitude of the applied signal ranged from 0 to 2 V_peak-to-peak_ in one second. If there are 100 digitized voltage increment steps, then there are 100 steps in one second for reaching 0 to 2 V_peak-to-peak_. The number of digitized voltage increment steps to reach each loading rate used in our experiment varied with 400 steps, 200 steps, 100 steps, and 20 steps. The dielectrophoretic force corresponding to the applied voltage was calculated in the following way. The DEP force is described by

(1) $${\vec{F}}_{total}=\sum_{0}^{\infty }-\nabla U_{n},\;U_{n}=-\frac{2\pi \varepsilon_{m}K_{n}r^{\left(2n+1\right)}}{(2n+1)!!}\sum_{i+j+k=n}\frac{1}{i!j!k!}{\left[\frac{\partial^{n}\Phi }{\partial x^{i}\partial y^{j}\partial z^{k}}\right]}^{2}\;K_{n}=\frac{n(2n+1)({\tilde{\varepsilon }}_{p}-{\tilde{\varepsilon }}_{m})}{n{\tilde{\varepsilon }}_{p}+(n+1){\tilde{\varepsilon }}_{m}}$$

where *n* is the force order, Φ refers to the electrostatic potential of the external electric field, and *K_n_* is the n^th^-order Clausius–Mossotti factor [14]. This equation was used to convert the voltage applied to the electrode into the DEP force. To do that, Matlab (R12, Mathworks) code was developed to calculate the total DEP force using Equation (1). The electrical-field profiles for the IDT electrodes used for the experiment, when the AC signal with 1 V_peak-to-peak_ and 1 MHz was applied to the electrodes, were generated from a finite element program (version 5.7, ANSYS Inc., Canonsburg, PA, USA) with a grid spacing of 0.2 μm. The experimental parameters and the generated electric-field data were used as inputs for the Matlab code.

### 2.5. Experimental Procedure of the Automatic System for DFS

For the measurement of Van der Waals interaction or hydrogen bond described in the Section 2.3, the following procedures was performed in the automatic system for DFS: (1) The prepared sample described in Section 2.3 were loaded into the system; (2) Variables such as voltage and frequency of input signal, ramping rate of voltage for loading rate, capture rate of image, and the format for saving data into the developed program described in Section 2.2 were set, the developed program was operated, and the control module and the measurement module described in Section 2.1 then automatically controlled the voltage and frequency of the input signal, the generation of the voltage ramping rate, and the measurement of the voltage and frequency of the input signal. Meanwhile, the observation module controlled the process of the capture rate of the image sequences that recorded the movement of the particles. (3) Lastly, we obtained time-lapsed image sequence from the CCD camera comprising the DEP movement of 100 particles and measured the data of the input signal corresponding to the applied voltage signal after performing the experiment procedures.

## 3. Results and Discussion

### 3.1. Synchronization of the Automatic System for DFS

**Figure 3:**
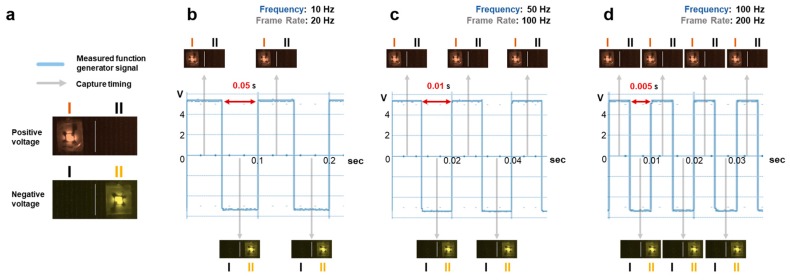
To confirm synchronization of the automatic system for DFS by using an LED circuit: (**a**) the LED circuit is operated by an input voltage applied to the function generator: the red LED (left LED) is at a positive voltage, and the yellow LED (right LED) is at a negative voltage. (**b**–**d**) The comparison between the applied signal with different frequencies and the captured images with the frame rates corresponding to the frequencies of the signal; (**b**) 10 Hz versus 20 frame/s, (**c**) 50 Hz versus 100 frame/s, and (**d**) 100 Hz versus 200 frame/s, where the amplitude of the applied signal was 10 V_peak-to-peak_.

In order to obtain reliable data from the automatic DEP force spectroscopy system, the measurement, observation, and control modules should be synchronized. To verify the synchronization of these modules, we provided 10 V_peak-to-peak_ values with three different frequencies, namely 10 Hz, 50 Hz, and 100 Hz, in the system, and the flip of the LED light was simultaneously captured by the CCD camera. As shown in Figure 3, the LED turns red (left LED) when a positive voltage is applied, while the LED turns yellow (right LED) for a negative voltage. The time for this change of colors of the LED light was matched to the frequency of the applied signal according to the reminding images. These results demonstrate that the synchronization of the three modules in the automatic DEP force spectroscopy system is achieved.

### 3.2. Verification of the Reliability and Stability of the Automatic System for DFS

**Figure 4:**
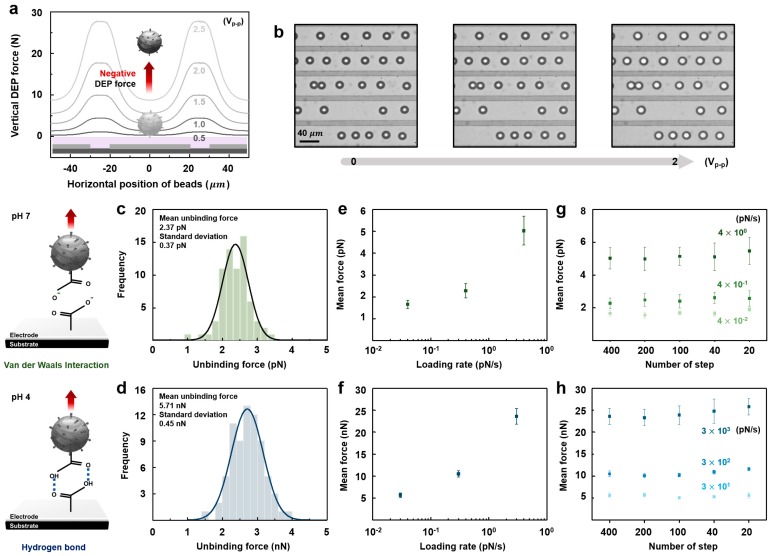
Numerical and experimental results: (**a**) numerical results of a DEP force and schematic illustration of the movement of the carboxyl-terminated molecule functionalized particle by a negative DEP force; (**b**) optical images of the movement of the arranged particles as the voltage increased from 0 V_peak-to-peak_ to 2 V_peak-to-peak_, (**c**,**d**) the measured unbinding forces: van der Waals interaction (**c**) and hydrogen bond (**d**); (**e**,**f**) the mean forces for a varying force loading rate: van der Waals interaction (**e**) and hydrogen bond (**f**); (**g**,**h**) the mean force with a varying ramping rate in combination with a force loading rate: van der Waals interaction (**g**) and hydrogen bond (**h**).

After this confirmation, the rupture forces of Van der Waals interactions and hydrogen bonds were measured under the same experimental conditions described in the previous report [14]. Figure 4a shows the calculated DEP forces when the IDT electrodes and the DEP parameters used for the experiment were applied into the simulation. In these simulation results, the carboxylate functionalized particle is moved toward the center of the electrode, which is covered by the TESPSA (also carboxyl-terminated molecules) functionalized silicon dioxide, and arranged on the center of the electrode by a negative DEP force. This arranged particle moves upward as the negative DEP force increases, as shown in Figure 4a. In the experiment, according to our numerical expectation, the carboxyl-terminated molecule functionalized particles were arranged through the center of the IDT electrodes covered by silicon dioxide with a negative DEP force, and the rupture event of the arranged particles then occurred, since the vertical negative DEP forcewas increased by the increasing applied AC voltage, as shown in Figure 4b (Van der Waals interaction in pH 7) [14]. Moreover, the unbinding force that allows rupture to occur is determined by the grayscale measurement method and the numerical simulation that has been already reported in Park et al. [14]. Figure 4c (Van der Waals interaction) and Figure 4d (hydrogen bond) show the experimental measured unbinding force using the automatic DFS system. Moreover, the mean forces that allow the rupture events to occur were also examined by varying the force loading rate. Figure 4e (Van der Waals interaction) and Figure 4f (hydrogen bond) are the results of this examination. Those results, as shown in Figure 4, correspond to the results in our previous report [14].

When an applied signal is increased for a certain loading period, the increased signal per loading period is generally assumed to be a linear input and the measurement result is assumed to be stable. However, this is not strictly true. Imagine a situation where we load 1 pN/s in the DEP system. Since the applied input signal in the system is digitized, 1 pN/s can be achieved with several different ramping rates. For example, this value can be reached from 0 to 1 pN by increasing one digitized step per second (e.g., the ramping rate to reach 1 pN/s is one per second) or by increasing 1000 digitized steps per second (e.g., the ramping rate to reach 1 pN/s is 1000 per second). These variations could affect the measurement results since the ramping rate is not linear [23,24]. We also examined the measurement stability of the DEP system when the ramping rate of the applied signal was changed. In this examination, we selected various force loading rates from the quasi-equilibrium (4 × 10^−2^ pN/s) to the high pulling event (3 × 10^3^ pN/s), where the mean rupture force has already been measured in Figure 4e,f, by varying the number of digitized force increment steps. Moreover, when the number of steps is 400 for 4 × 10^−2^ pN/s, as shown in Figure 4g,h, the ramping rate to reach 4 × 10^−2^ pN is 400 steps per second. This result clearly shows that the measurement results are stable in the range where the ramping rate was from 400 steps to 20 steps. It should be stated that the ramping rate cannot be decreased in our DEP system because the minimum ramping rate of the voltage increment is for up to 20 steps in our system. Further experimentation is needed to confirm the ramping rate issue below 20 steps.

## 4. Conclusions

In conclusion, we developed three modules including the measurement module, observation module, and the control module for an automatic controller DEP tweezers-based force spectroscopy system in a microfluidic device. All three modules were automatically controlled via a LabVIEW program with a GUI interface. We also showed that the modules are clearly synchronized when they are working. Furthermore, the measurement stability of the system responding to the ramping rate when the applied signal is increased for the certain time was also verified. Lastly, the rupture forces of the weak binding interactions were measured by the developed system and compared to the previous results. We verified the reliability and stability of the automatic control DEP tweezers-based force spectroscopy system. We expect that the developed system will be useful for investigating the weak binding interactions in accurate, simple, powerful, and inexpensive platforms.
